# Amputation of the Arm by Means of the Elastic Ligature

**Published:** 1877-01

**Authors:** 


Amputation of the Arm by Means of the Elastic Ligature. In the *Lyon Medicale* this operation is recorded as performed by Prof. O. G. Silvestri, of Vicenza. Surgeons naturally hesitate to perform resection or amputation in cases of white swelling of the knee or elbow. The process not being arrested on account of inadequate remedial measures, the patient loses strength, and becomes extremely emaciated; it is at this period of the disease that the operation is usually performed, though the general condition of the patient would almost contraindicate any active interference.

Silvestri, who first introduced the elastic compression known under the name of "Esmarch's method," has proposed the employment of the elastic ligature in the above cases, and has published a case in which the result was most gratifying. It was that of a young man, twenty-two years old, of a scrofulous constitution, who for six months had suffered from caries of the sixth, seventh, eighth, and ninth ribs, in their convexities; there was complete caries of the left elbow-joint, and the right hand was threatened with the same condition. There were high fever, colliquative sweats, and diarrhœa, which would yield to no treatment; absolute anorexia, intense pains in the elbow, and extreme emaciation. Though the condition of the elbow-joint indicated an operation, the feebleness of the patient contraindicated it. But, as the patient was urgent to have something done, Silvestri, with the consent of his colleagues, resolved to apply the elastic ligature.

On the 8th of May, 1874, accordingly, the patient's arm, below the insertion of the deltoid, was enveloped with a gum-elastic band, about two millemetres in diameter, and covered with silk thread. Twenty turns of the band were made, the latter being always kept in its greatest extension, and the two ends were tied with a silk band. The patient received seven and a half grammes of chloral, which produced sleep. No pain was experienced. The pressure exercised, calculated according to the elasticity of the band, was equal to twenty-one kilogrammes at each point, consequently forty-two kilogrammes for the whole diameter. The pulse, at the time of operation, was 100; five hours after, 112; and six hours after, 100. There was no fever on the following day; the sweats and diarrhœa ceased, and the appetite returned. Milk diet was ordered, under which the patient soon began to gain flesh.

Gradually the bands penetrated the soft tissues, and at the same time lost thier parallelism. The circumference of the arm, where the bands were applied, was eighteen centimetres at the time of operation; four days after it was eleven centimetres; six days after, ten and a half centimetres, and ten centimetres on the 26th of May. On the evening of May 29th it was found to be nine and one-quarter centimetres, and on June 3rd it was reduced to eight centimetres.

On June 18th the arm and bands fell off spontaneously, the process having lasted forty days. The stump, in its upper portion, had cicatrized. The remaining portion was dressed with dry lint. The further course of the case was favorable.

The author draws the following conclusions :

1. The compression exercised intercepts all communication between the limb and the rest of the body; the morbid material from the seat of the disease cannot, therefore, enter the circulation; furthermore, drainage from the morbid *foyer* ceases.

2. There is no loss of blood.

3. Cicatrization takes place slowly, and the patient bears it easily.

4. The patient's forces are economized.

The author does not hesitate to employ this method in all those cases where the general condition of the patient offers no prospect of success to the performance of a bloody operation.—*N. Y. Medical Journal.*

